# LINC00311 is overexpressed in ankylosing spondylitis and predict treatment outcomes and recurrence

**DOI:** 10.1186/s12891-019-2647-4

**Published:** 2019-06-07

**Authors:** Hongfa Zhong, Mingliang Zhong

**Affiliations:** Department of orthopaedics, Ganzhou People’s Hospital, No.16 Meiguang Avenue, Ganzhou City, Jiang Xi Province 341000 People’s Republic of China

**Keywords:** Ankylosing spondylitis, lncRNA LINC00311, Re-hospitalization

## Abstract

**Background:**

LncRNA LINC00311 participates in osteoporosis, which shows inverse pathological changes to ankylosing spondylitis (AS), indicating that LINC00311 is also involved in AS.

**Methods:**

All the participants were enrolled in Ganzhou People’s Hospital between January 2016 and January 2018 after this study was approved by Ganzhou People’s Hospital Ethics Committee. Disease activity determination, follow-up and RT-qPCR were carried out during the research.

**Results:**

In the present study we found that LINC00311 was upregulated in AS patients comparing to healthy controls, and upregulation of LINC00311 distinguished AS patients from healthy controls. LINC00311 expression levels were positively correlated with disease activity. Comparing to pre-treatment levels, LINC00311 expression level decreased significantly after treatment. During 2-year follow-up, patients with high levels of LINC00311 showed a significantly higher rate of rehospitalization.

**Conclusions:**

Therefore, LINC00311 is overexpressed in AS and predict treatment outcomes and recurrence.

## Introduction

Ankylosing spondylitis (AS) is a type of chronic inflammatory rheumatic disease that mainly affects skeletal system [1]. AS not only causes chronic back pain but may also result in functional and structural impairments [1]. As a common type of clinical disorder AS affects about 1 out of 110 people in western countries and about 1 out of 200 people in China [2, 3]. In spite of the efforts made on the treatment of AS, prognosis of AS is poor and most AS patients will experience recurrence within a short-term after discharge [4, 5]. Therefore, accurate prognosis is needed to predict the development of AS.

Genetic factors, such as the altered gene expression, contribute to the development of progression of AS [6, 7]. Besides protein players, non-coding RNAs, such as long non-coding RNAs (> 200 nt, lncRNAs) have also been characterized as critical regulators in human disease development and progression due to their roles in regulating gene expression at multiple levels [8, 9]. Analysis of the expression pattern and functions of lncRNAs may contribute to disease treatment and prediction [8, 9], while function of most lncRNAs remains unclear. A recent study reported that lncRNA LINC00311 participates in osteoporosis by regulating the differentiation and proliferation of osteoclasts [10]. It is known that osteoporosis and AS have inverse pathological changes [11]. Our study therefore was carried out to investigate the involvement of LINC00311 in AS.

## Methods

### Research patients

Patient group in this study included 80 AS patients (47 males and 33 females, 24 to 46 years, 30.3 ± 4.8 years). Inclusion criteria: 1) newly diagnosed AS patients; 2) no therapies initiated before admission. Exclusion criteria: 1) other clinical disorders were observed; 2) with previous history of malignancies; 3) with family history of malignancies. Control group included 80 healthy volunteers (48 males and 32 females, 23 to 47 years, 32.1 ± 4.9 years) and 22 low back pain (LBP) patients (12 males and 10 females, 24 to 49 years, 31.9 ± 5.1 years). Controls and LBP patients were included to match the distributions of age and gender of AS patient group. Controls with previous or family history of malignancies were excluded. All the participants were enrolled in Ganzhou People’s Hospital between January 2016 and January 2018 after this study was approved by Ganzhou People’s Hospital Ethics Committee. All patients and controls signed written informed consent.

### Disease activity determination

Both Ankylosing Spondylitis Disease Activity Index (BASDAI) and The Ankylosing Spondylitis Disease Activity Score (ASDAS) 1–4 were used to determine the disease activity of all 80 AS patients. Levels of inflammatory markers C reactive protein (CRP) and Estrogen Receptor (ESR) in plasma were measured with ELISA using Human C Reactive Protein ELISA Kit (CRP) (ab99995, Abcam) and human Estrogen Receptor Alpha ELISA Kit (ab128499, Abcam), respectively.

### Plasma and treatment

Before any therapies were initiated, blood (5 ml) was extracted from each participant under fasting conditions. Blood was centrifuged at room temperature for 2 h at 1200 g to prepare plasma samples.

All patients (AS and low back pain) were treated with nonsteroidal anti-inflammatory drugs, such as indomethacin and naproxen (doses varies across patients based on their disease conditions). The relieved pain, inflammation and stiffness indicated the relieved disease conditions (reflected by BASDAI and ASDAS scores).

At 2 months after the initiation of treatment (significantly improved BASDAI and ASDAS were observed at this time point), blood (5 ml) was extracted from each patient under fasting conditions to prepare plasma samples using aforementioned methods.

### Follow-up

The 80 AS patients were monitored for 2 years after admission. Follow-up was performed through phone call or outpatient visit. The cases of re-hospitalization were recorded and analyzed.

### RT-qPCR

Plasma samples were mixed with Ribozol reagent (Thermo Fisher Scientific) to extract total RNAs. Total RNAs were used as template to perform reverse transcriptions using First Strand cDNA Synthesis Kit for RT-PCR (AMV, Sigma-Aldrich, USA). To detect the expression of LINC00311, qPCR reaction mixtures were prepared using Invitrogen SuperScript® III Platinum® SYBR® Green One-Step qRT-PCR Kit (Thermo Fisher Scientific) with 18S rRNA as endogenous control. All PCR reactions were performed 3 times and data were normalized using 2^-ΔΔCT^ method.

### Statistical analysis

Mean values presented in this paper were from 3 biological replicates of each experiment. Differences among 3 groups were analyzed by performing ANOVA (one-way) and Tukey test. Differences between pre- and post-treatment levels of LINC00311 in AS patients were analyzed by performing paired test. Diagnostic values of LINC00311 for AS were analyzed by performing ROC curve analyses. In ROC curve analysis, AS patients were true positive controls and healthy controls were true negative controls. Comparisons of re-hospitalization rates were analyzed by performing Chi-squared test. Correlations were analyzed by performing linear regression analysis. Statistically significant level was *p* < 0.05.

## Results

### Plasma LINC00311 was upregulated in AS

**Fig. 1:**
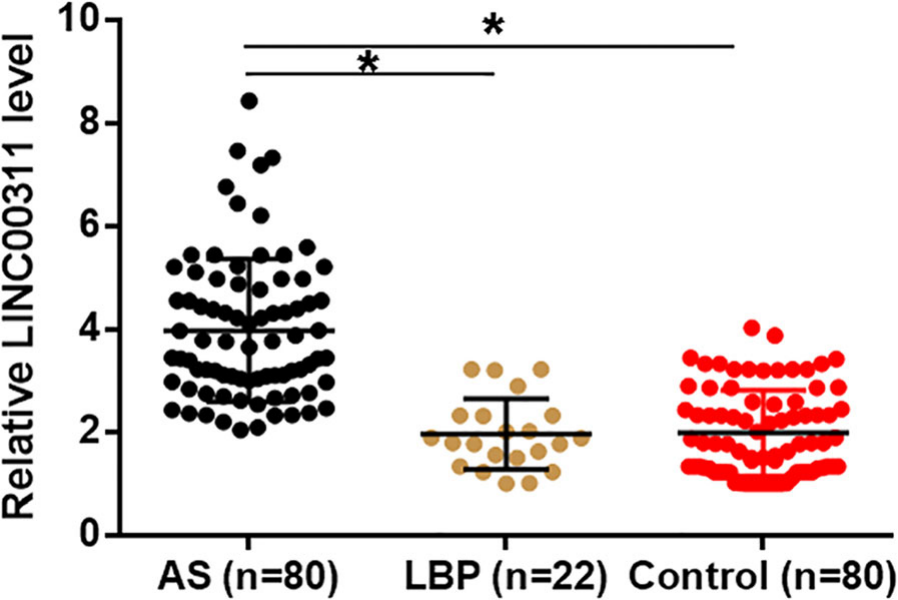
Plasma LINC00311 was upregulated in AS. LINC00311 in plasma of was detected by RT-qPCR and expression data were compared by performing ANOVA (one-way) and Tukey test. It was observed that plasma levels of LINC00311 were significantly higher in AS patients than in controls and LBP patients (*, *p* < 0.05)

LINC00311 in plasma of AS patients (*n* = 80), LBP patients (*n* = 22) and controls (*n* = 80) was detected by performing RT-qPCR experiments. Plasma levels of LINC00311 were compared by ANOVA (one-way) and Tukey test. It was observed that plasma levels of LINC00311 were significantly higher in AS patients comparing to controls and LBP patients (Fig. 1, *p* = 0.012 and 0.011, respectively). This finding suggest that altered expression of LINC00311 is likely involved in the pathogenesis of AS.

### Upregulated plasma levels of LINC00311 showed diagnostic values for AS

**Fig. 2:**
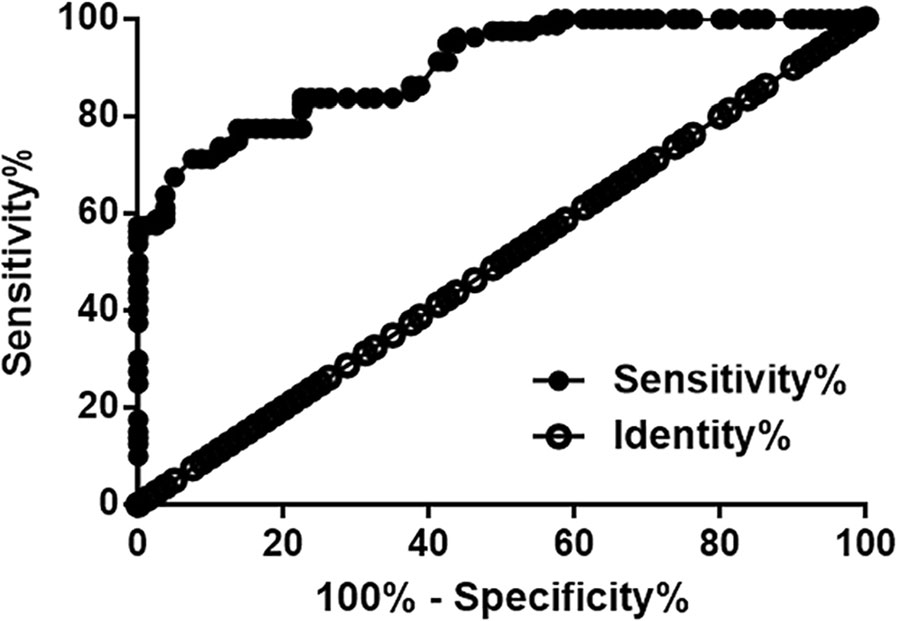
Upregulated plasma levels of LINC00311 showed diagnostic values for AS. ROC curve analysis showed that upregulated plasma levels of LINC00311 distinguished AS patient from healthy controls

Diagnostic values of LINC00311 for AS were analyzed by performing ROC curve analyses. In ROC curve analysis, AS patients were true positive controls and healthy controls were true negative controls. As shown in Fig. 2, area under the curve was 0.9041, with standard error of 0.02268 and 95% confidence interval of 0.8569–0.9459 (Fig. 2, *p* < 0.0001). Therefore, plasma LINC00311 may be a potential diagnostic marker for AS.

### LINC00311 expression levels were positively correlated with disease activity

BASDAI and ASDAS 1–4 were widely used to determine the activities of AS. In addition, levels of CRP and ESR are also used to reflect the inflammation in AS patients. In this study all patients were subjected to BASDAI and ASDAS 1–4 scoring and plasma levels of CRP and ESR in AS patients were measured by performing ELISA. Linear regression was performed to analyze the correlations between those markers and plasma levels of LINC00311. As shown in Table 1, BASDAI and ASDAS 1–4 were significantly and positively correlated with CRP and ESR (R square > 0.65, all *p* values < 0.0001). In addition, BASDAI, ASDAS 1–4, CRP and ESR were also significantly and positively correlated with plasma levels of LINC00311 (R square > 0.65, all *p* values < 0.0001).

**Table 1:** LINC00311 expression levels were positively correlated with disease activity (Linear regression of BASDAI, ASDAS 1-4, CRP and ESR were also significantly and positively correlated with plasma levels of LINC00311)

Markers	Cases	ESR		CRP		LINC00311	
		R square	*p* value	R square	*p* value	R square	*p* value
BASDAI	80	0.89	< 0.0001	0.91	< 0.0001	0.86	< 0.0001
ASDAS 1	80	0.89	< 0.0001	0.88	< 0.0001	0.87	< 0.0001
ASDAS 2	80	0.90	< 0.0001	0.88	< 0.0001	0.84	< 0.0001
ASDAS 3	80	0.93	< 0.0001	0.92	< 0.0001	0.84	< 0.0001
ASDAS 4	80	0.89	< 0.0001	0.93	< 0.0001	0.85	< 0.0001
ESR	80	1.00	< 0.0001	0.94	< 0.0001	0.84	< 0.0001
CRP	80	0.94	< 0.0001	1.00	< 0.0001	0.89	< 0.0001

### LINC00311 levels in plasma decreased after treatment

**Fig. 3:**
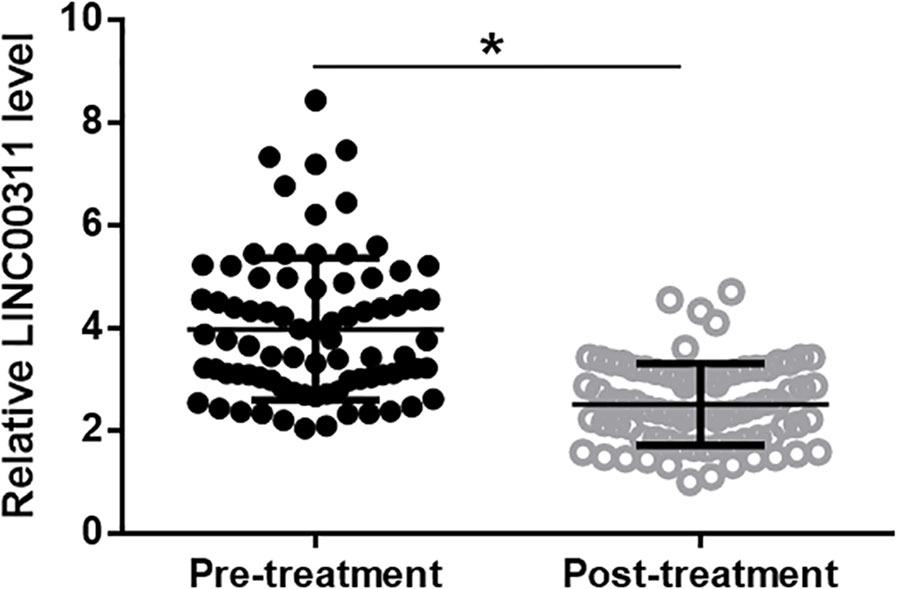
LINC00311 levels in plasma decreased after treatment. Plasma levels of LINC00311 were compared by performing paired t test between 2 time points. It was observed that, comparing to pre-treatment levels, plasma levels of LINC00311 were significantly reduced at 2 months after the initiation of treatment (*, *p* < 0.05)

Plasma levels of LINC00311 in AS patients were measured both before and 2 months after the initiation of treatment. Plasma levels of LINC00311 were compared by performing paired t test between 2 time points. Comparing to pre-treatment levels, plasma levels of LINC00311 were significantly reduced at 2 months after the initiation of treatment (Fig. 3, *p* = 0.017).

### Plasm levels of LINC00311 before treatment predicted re-hospitalization

**Fig. 4:**
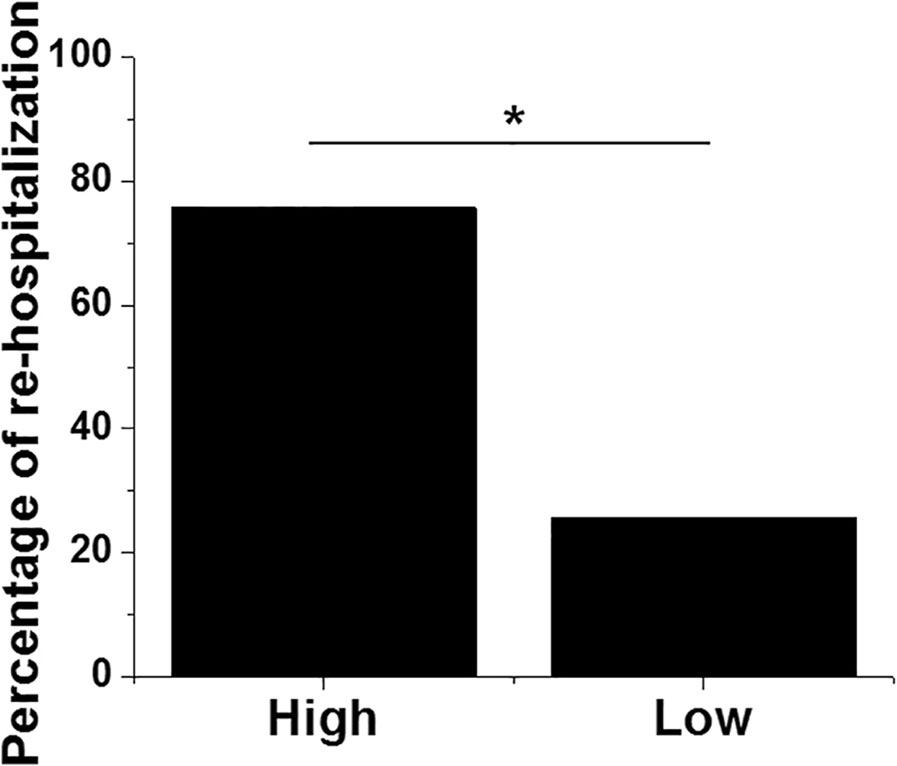
Plasma levels of LINC00311 before treatment predicted re-hospitalization. Chi-squared test showed that patients with high levels of LINC00311 had a significantly higher rate of re-hospitalization (*, *p* < 0.05)

The 80 patients were divided into high (*n* = 37) and low (*n* = 43) LINC00311 level groups according to Youden’s index. During the 2-year follow-up, 39 patients were re-hospitalized, including 28 cases in high LINC00311 level group (75.7%) and 11 cases in low LINC00311 level group (25.6%). Comparisons of re-hospitalization rates were analyzed by performing Chi-squared test. It was observed that patients with high levels of LINC00311 showed a significantly higher rate of re-hospitalization (Fig. 4, chi-square statistic =19.9751, *p* < 0.011).

## Discussion

We analyzed the expression pattern of LINC00311 in AS and also explored the clinical significance of altered plasma levels of LINC00311 for AS. We found LINC00311 can be used to assist the diagnosis and prognosis of AS.

Previous studies have identified quite a few lncRNAs related to AS. Li et al. reported that lncRNA AK001085 was downregulated in AS, and the downregulation of AK001085 effectively separated AS patients from healthy controls [12]. In another study, Lan et al. found that the reduced expression of lncRNA TUG1 was closely correlated with the course of treatment and disease activity of AS patients [13]. In a recent study, lncRNA MEG3 was found to be downregulated in AS and is associated with hospitalization time, disease activity and disease duration [14]. A recent study reported that lncRNA LINC00311 participates in osteoporosis [10], which has inverse pathological changes to AS [11], indicating the potential involvement of LINC00311 in AS. In the present study we proved that LINC00311 was upregulated in AS and may serve as a potential diagnostic biomarker for AS. However, the expression pattern of LINC00311 in other human diseases is unknown, so the sensitivity and specificity are still questionable.

BASDAI and ASDAS were widely used to asses AS activity [15]. In addition, levels of CRP and ESR are also used to reflect the inflammation in AS patients [16, 17]. In the present study we showed that BASDAI and ASDAS 1–4 were significant and positively correlated with plasma levels of CRP and ESR in AS patients, indicating that BASDAI and ASDAS can be used to reflect the in vivo inflammation conditions of AS patients. In addition, plasma levels of LINC00311 were also significantly and positively correlated with BASDAI and ASDAS scores as well as plasma levels of CRP and ESR. Therefore, plasma LINC00311 may serve as a new biomarker of AS activity.

Besides diagnostic potentials and the correlation with disease acidity, our study also focused on re-hospitalization, which is extremely common among AS patients [7]. We found that high plasma levels of LINC00311 were accompanied by high re-hospitalization rate. Therefore, measurement of plasma levels of LINC00311 before treatment may be used to predict its recurrence, and individualized treatment strategies may be developed based on plasma levels of LINC00311 to reduce the recurrence rate.

## Conclusion

In conclusion, LINC00311 was upregulated in AS, and upregulated LINC00311 has clinical potentials for the diagnosis and prognosis of AS.

## Data Availability

The datasets used and/or analyzed during the current study are available from the corresponding author on reasonable request.

## Abbreviations

AS: Ankylosing spondylitis
ASDAS: Ankylosing Spondylitis Disease Activity Score
BASDAI: Bath Ankylosing Spondylitis Disease Activity Index
CRP: C reactive protein
ESR: Estrogen Receptor
lncRNAs: Long non-coding RNAs
